# Mirror, mirror on my screen: Focus on self-presentation on social media is associated with perfectionism and disordered eating among adolescents. Results from the “LifeOnSoMe”-study

**DOI:** 10.1186/s12889-024-19317-9

**Published:** 2024-09-10

**Authors:** Hilde Einarsdatter Danielsen, Turi Reiten Finserås, Amanda Iselin Olesen Andersen, Gunnhild Johnsen Hjetland, Vivian Woodfin, Jens Christoffer Skogen

**Affiliations:** 1 Department of Health Promotion, Norwegian Institute of Public Health, Bergen, Norway; 2 Department of Clinical Psychology, University of Bergen, Bergen, Norway; 3 Centre for Evaluation of Public Health Measures, Norwegian Institute of Public Health, Oslo, Norway; 4 Department of Clinical Psychology, Solli District Psychiatric Centre, Bergen, Norway; 5 Center for Alcohol and Drug Research (KORFOR), Stavanger University Hospital, Stavanger, Norway

**Keywords:** Adolescents, Self-presentation, Upward social comparison, Social media, Perfectionism, Disordered eating

## Abstract

**Background:**

Social media use, perfectionism, and disordered eating have all increased over the last decades. Some studies indicate that there is a relationship between self-presentation behaviors and being exposed to others’ self-presentation on social media, and disordered eating. Studies also show that the relationship between focus on self-presentation and highly visual social media is stronger than for non-visual social media, hence facilitating upward social comparison. Nevertheless, no previous studies have investigated the link between adolescents’ *focus* on self-presentation and upward social comparison on social media, and perfectionism and disordered eating, which is the aim of the present study.

**Method:**

The present study is based on a cross-sectional survey from the “LifeOnSoMe”-study (*N* = 3424), conducted in 2020 and 2021. Respondents were high school students (mean age 17.3 years, 56% females) in Bergen, Norway. Multiple regression analysis was performed, where SPAUSCIS, a measure of self-presentation and upward social comparison, was the independent variable. Perfectionism and disordered eating were dependent variables. Self-reported age, gender, and subjective socioeconomic status were used as covariates, as well as frequency and duration of social media use. Regression models were performed to compare proportions across the median split of SPAUSCIS.

**Results:**

The multiple regression analysis showed that increased focus on self-presentation and upward social comparison on social media were positively associated with both perfectionism (standardized coefficient 0.28) and disordered eating. A stronger association for girls than boys was found for disordered eating (standardized coefficient 0.39 for girls and 0.29 for boys). There was no gender moderation for perfectionism.

**Conclusions:**

Findings suggest that focus on self-presentation and upward social comparison on social media is associated with perfectionism and disordered eating. We recommend promoting a healthy use of social media. This could be established by increasing adolescents’ ability to reflect on and think critically about self-presentation and upward social comparison on social media.

## Introduction

Growing up today means growing up in a highly digitalized world where social media and online communication plays an important role in adolescents’ lives. Social media can be defined as “highly interactive platforms via which individuals and communities share, co-create, discuss, and modify user-generated content” [1, pp. 241]. Previous studies have largely focused on the temporal aspects of social media use, and some studies indicate that social media use is associated with more mental health problems and decreased well-being [2]. For example, there are reports that more time spent on social media is associated with symptoms of depression and anxiety [3, 4], sleep issues [3, 5], and body dissatisfaction [6]. However, not all research confirms these associations [7, 8], and recent studies have indicated that the observed link between time spent on social media and mental health is too small to be of practical importance [9]. A recent longitudinal study found time spent on social media to be the least important factor in relation to adolescent mental health [10]. Nevertheless, there is an ongoing and almost ubiquitous concern regarding social media’s potential negative effect on mental health. Considering this, it is increasingly recognized that it is important to investigate more than adolescents’ time spent on social media, such as their usage patterns. After all, social media offers a range of opportunities, such as seeking out like-minded others or specific topics and inspiration, for example, for food, fitness, and a healthy lifestyle. Although inspirational hashtags and pictures may be positive to many adolescents, they also frequently present a “perfect” lifestyle and some of them could even be considered unhealthy inspirations.

### Self-presentation

Self-presentation on social media has been highlighted as potentially important in connection with mental health and well-being among adolescents [e.g. 11–14]. Baumeister & Hutton [15] defined self-presentation as an individual practice related to how one presents oneself to others, motivated by a wish to make a socially desirable impression on others, and simultaneously, stay true to one’s beliefs and ideals. On social media, self-presentation may include presenting and sharing self-made content, posting of personal opinions, sharing online content of interest, and “selfies” and pictures [14, 16]. An American report noted that adolescents are more engaged in self-presentation activities on social media than any other age group [17]. As increased independence from parents is an important developmental milestone for adolescents, external validation from others may be particularly important for this age group [18]. Feedback on social media posts through likes and comments, may therefore be an important source of external validation from peers. Considering this, it is likely that many adolescents put great importance on how they present themselves on social media. In addition, social media is a suitable arena for self-presenting activities, as it gives the adolescent control over what, when and how to present themselves on the platform of their choosing [12]. Functions such as likes, comments, followers [19], and other measures of engagement, which are implemented on many social media platforms in one form or another, give ample opportunity for immediate feedback on posted content. Hence, this provides cues of social desirability and direction to align future social media posts with how the adolescents prefer to present themselves on these platforms [12]. These features of social media, in addition to the ability to reach a large and varied audience, may serve to facilitate self-presentation [20].

Self-presentation behaviors [e.g. 13, 14] on social media are closely connected to *focus on self-presentatio*n [12, 21, 22]. Focus on self-presentation consist of caring about how you present yourself on social media, e.g., retouching pictures before posting them, caring about having a nice social media feed or striving for positive feedback on your social media posts, and can be independent of how much or how often a person post something [12, 21]. As such, focus on self-presentation differs from self-presentation behaviors, which have been more extensively researched [e.g. 13, 14]. A study showed that many adolescents have a desire to focus less on their self-presentation on social media, but that they think it is hard to resist the pressure of having a good feed and receiving positive feedback such as likes, comments, and followers [23]. A higher focus on self-presentation has been linked to the use of highly visual social media platforms like Instagram, TikTok and Facebook, rather than less visual platforms [12].

Likewise, use of social media has been linked to more social comparison, and in particular upward social comparison [24, 25]. Social comparison is the propensity to compare one’s characteristics to other people to obtain information about how we are doing relative to others [26]. *Upward social comparison* occurs when one compares oneself to someone perceived as better or with higher status than oneself, which may be especially prevailing on social media. One study found that social media users mostly presume that other users have better lives than themselves [27]. Moreover, following a large number of people on social media increases the reference group to which adolescents compare themselves, and may include high-status people like “influencers” and celebrities [28]. Upward social comparison has been reported to be associated with more negative feelings such as depression and lower life satisfaction [11, 29], and more body dissatisfaction [30]. Hawes et al. [31] also found that preoccupation with appearance comparison on social media was linked to symptoms of anxiety and depression among adolescents. Thus, while self-presentation on social media may not be harmful, feedback-seeking and upward social comparison may be damaging to mental health.

### Perfectionism

In addition to being a central period for self-presentation activities, adolescence seems to be a particularly susceptible period for the development of perfectionism. Perfectionism is a personality disposition that may be defined as the tendency to set unrealistically high performance standards and striving for flawlessness [32]. Perfectionism is thought to be a disposition largely consolidated in adolescence as a part of a general identity formation [18].

Over the last 30 years, there has been an increase in perfectionistic personality traits among young adults [33]. Curran & Hill [33] hypothesize that this might be a consequence of the rise of a competitive cultural trend, and also the advent of social media in young peoples’ lives. As social media gives adolescents control over how they self-present, social media also allows them to create a (highly) specific and “ideal” image of themselves. Considering these perspectives, Curran & Hill [33] suggest that young people perceive their social context as more demanding and subsequently believe others will evaluate them more harshly. An experimental study investigating the effect of selfie taking and posting on social media on women’s mood and body image, concluded that the psychological states subsequent of posting the selfies, was related to self-consciousness and/or fear of being negatively evaluated [14]. Thus, adolescents of today may to a larger extent strive for perfectionistic self-presentation in order to secure acceptance among peers than older generations. Hewitt et al. [34] suggested the concept of perfectionistic self-presentation and argued that this is a maladaptive self-presentation style. One facet of perfectionistic self-presentation is perfectionistic self-promotion, which includes proclaiming and displaying one’s perfection [34]. Through features such as likes, comments and followers, social media may be a key arena for perfectionistic self-presentation and self-promotion, and hence a way of seeking external validation and approval in a socially acceptable way among adolescents.

A study found that perfectionistic concerns predicted longitudinal change in self-presentation and that perfectionistic self-presentation was linked to decreased well-being [35]. Hence, perfectionistic concerns indirectly affected subjective well-being through self-presentation [35]. Perfectionistic self-presentation also predicted changes in both positive and negative affect [35]. In a meta-analysis, perfectionism was found to be positively associated to different psychological disorders and symptoms, including body dissatisfaction, and eating disorders [36].

### Disordered eating

Previous research has linked disordered eating to self-presentation [25] and to perfectionism [36–38]. A person with disordered eating will be obsessed with food and have constant thoughts about eating, body shape, weight, and food. Symptoms of disordered eating above a certain level may constitute an eating disorder according to the criteria in the Diagnostic and Statistical Manual of Mental Disorders (DSM 5th Ed.) [39] and the International Classification of Mental and Behavioral Disorders (ICD-10) [40]. A meta-analysis reported that over the last 20 years, there has been an increase in the weighted means of point eating disorder prevalence from 3.5% for the years 2000–2006 to 7.5% for the years 2013–2018 [41]. The prevalence for eating disorder was consistently higher among women compared to men regardless of timeframe (lifetime, 12-months, point prevalence). In the same meta-analysis, the authors also stressed the finding that eating disorders are highly prevalent in adolescence, with an estimated point prevalence between 6% and 8% [41].

As a great deal of content on social media promotes pictures of healthy food, diets, exercise, and appearance-focused images and idealized bodies, concerns have been raised that social media may contribute to body image concerns and disordered eating, especially among adolescents [42, 43]. A systematic review, conducted by Holland & Tiggemann [43] showed that exposure to content on Facebook, in particular photo-based activity, was positively associated with negative body image and disordered eating behaviours in children, adolescents, and young adults. Another study found similar results; more exposure to appearance-related pictures on Facebook was associated with self-objectification, weight dissatisfaction, thin ideal internalization, and drive for thinness among girls [44].

Similarly, research indicates that exposure to others’ “perfect” self-presentations on social media may reinforce one’s own body image concerns and disordered eating [24, 25]. Fardouly et al. [24] investigated young adult women’s appearance comparisons in different contexts in everyday life. They found that most of the appearance comparisons were made in person and on social media, and that the participants made relatively more upward appearance comparisons on social media than in person. They also found that upward appearance comparisons made on social media were associated with more body dissatisfaction than in person. In addition, upward appearance comparisons on social media yielded more thoughts about dieting than in person comparisons, but no difference in the likelihood of dieting-behaviours [24].

Furthermore, Rodgers et al. [25] found that social media use was positively correlated with higher internalization of appearance ideals, including a higher tendency to engage in appearance comparison, body dissatisfaction, muscle change behaviours and dietary restraints among both boys and girls. In addition, the internalization of social media ideals, the muscular ideals and appearance comparisons, were positively associated with body dissatisfaction, muscle change behaviours and dietary restraints. Other research has reported similar results [6, 45]. Mclean et al. [45] found for instance, that self-presentation on social media was associated with internalization of social media ideals, and that the internalization mediated the effect of social media on appearance upward comparison and body dissatisfaction. A scoping review conducted by Dane & Bhatia [46] also reported that in cases where social media use led to eating disorder, the thin/fit body ideal internalization and social comparison often functioned as mediating pathways.

### Theoretical framework, summary and the current study

The Tripartite Influence Model (TIM) may serve as a theoretical framework linking the concept of focus on self-presentation and upward social comparison on social media, with perfectionism and disordered eating [47]. The Tripartite Influence Model is a framework that can be used when exploring the relationship between social media use and body dissatisfaction. It proposes that pressures from peers, family and media makes one conform to certain appearance ideals, which can lead to internalization of body ideals, followed by physical appearance comparison with others [48]. This study’s focus on self-presentation and upward social comparison on social media, aligns with the Tripartite Influence Model’s emphasis on how media and peers (e.g. to what content that receives positive feedback from peers), may contribute to adolescents’ perception of ideal body standards. Findings indicate that higher focus on self-presentation is more strongly linked to visual social media platforms than less visual platforms [12]. This support the Tripartite Influence Model theory that media pressure, especially through highly visual social media, leads to increased body ideal internalization an upward comparison with others. Additionally, the association between social media use and disordered eating can be understood through pressure to conform to societal ideals, such as body ideals, as proposed in the Tripartite Influence Model. Perfectionism, which is linked to disordered eating [36–38], may be driven by similar societal pressures.

Research on adolescents’ use of social media is increasingly shifting focus away from looking merely at time spent to include potential consequences of specific aspects of adolescents’ social media usage patterns [2]. The use of social media, perfectionism, and disordered eating have all increased over the last decades [33, 41, 49]. Studies indicate a relationship between being exposed to how others present themselves on social media and body dissatisfaction and disordered eating [24, 25, 43], and some studies have also investigated the relationship between self-presentation behaviors and body dissatisfaction [13, 14, 30]. Moving beyond self-presentation behaviors, such as the frequency or content of social media posts, one study showed that being preoccupied with appearance on social media, was associated with increased risk for problems like appearance related anxiety and disordered eating [22]. In two previous studies, we showed that preoccupation with likes and comments, retouching photos of oneself, deleting photos with too few likes, and upward social comparison, collectively referred to as “focus on self-presentation”, was associated with more symptoms of anxiety and depression [12] and that focus on self-presentation varied significantly between adolescents [21].

Hence, the aim of the present study is to investigated the link between focus on self-presentation on social media, and perfectionism and disordered eating. Based on previous studies we hypothesize that focus on self-presentation and upward social comparison is positively associated with (i) perfectionism and (ii) disordered eating, and (iii) self-reported diagnosis of an eating disorder.

## Materials and methods

### Study sample

This study is based on data from the “LifeOnSoMe” study carried out at public senior high schools in Bergen, Norway. Pupils aged 16 or older were invited to participate, giving an age range from 16 to 21 years old. Information about the survey was conveyed both by the teacher and digitally. The online web survey was conducted digitally. One school hour was set aside for carrying out the survey. The total number of eligible participants was 3,424 (mean age was 17.3 years (standard deviation 1.0)), and 56% (*n* = 1916) of the participants were girls. This study included data from two survey waves conducted in September-October 2020 and June-September 2021. For participants who responded in both waves, only their 2020 responses were used in this analysis. The response rate was 53% in 2020 and 35% in 2021. The research data was stored on secure storage facilities located at the Norwegian Institute of Public Health, which prevent the authors from providing the data as supplementary information, according to the General Data Protection Regulation (GDPR). Only researchers with approval from the Regional Ethical Committee had access. The study was approved by the Regional Ethical Committee, and is in accordance with the General Data Protection Regulation. Additional information about the study is available elsewhere [23, 50].

### Variables

#### Self-reported sociodemographics

The participants reported their age, gender, and subjective socioeconomic status. A small proportion of the participants did not state their age (*n* = 157). For gender, participants could choose between three options: “girl”, “boy”, and “other/non-binary”. Because too few participants (< 50) answered “other/non-binary”, these were excluded from the data set due to privacy concerns. Relative socioeconomic status was assessed by asking the participants to estimate how economically well off their families are compared to others, ranging from «very poor» (scored 0) to «very well off» (scored 10).

#### Amount of social media use

Two questions were included related to social media use in general: “How often do you use social media?” and “On the days that you use social media, approximately how much time do you spend on social media?”, giving an estimate of the frequency and duration of their usage, respectively. For frequency, the response alternatives were “almost never”, “several times a month, but rarer than once a week”, “1–2 times per week”, “3–4 times per week”, “5–6 times per week”, “every day”, “several times each day”, and “almost constantly”. In the present study, we differentiated between “daily or less”, “many times a day”, and “almost constantly”. For duration, seven response alternatives ranging from “less than 30 min” to “more than 5 h” were available. In the present study, we differentiate between “<2 h”, “2–4 h”, “>4–5 h”, and “>5 h”.

#### Independent variable: Self-Presentation and Upward Social Comparison Inclination Scale (SPAUSCIS)

The items used to assess upward social comparison and aspects of self-presentation were developed based on focus group interviews with senior high school pupils [23], and have been shown to have adequate psychometric properties in both this sample [21] and elsewhere [12]. Cronbach’s $\alpha$was 0.87, indicating a very good internal consistency. The results of an exploratory factor analysis (EFA) and a confirmatory factor analysis (CFA) for the SPAUSCIS have been reported in a previous publication based on the “LifeOnSoMe”-data [21]. Also, EFA and CFA was investigated in another, smaller sample of senior high school students [12]. The results from both studies strongly suggested a unidimensional scale and the fit indices from CFA were all considered good. Examples of items included in SPAUSCIS are “I retouch images of myself to look better before I post them on social media”, “I use a lot of time and energy on the content I post on social media”, and “The response I get for what I post (images/status updates/stories) impacts how I feel”. The response categories were “not at all”, “very little”, “sometimes/partly true”, “a lot”, and “very much”, coded 1–5. The mean summed score thus ranges from 1 to 5, with higher scores indicating a higher focus on self-presentation and upward social comparison on social media.

#### Dependent variables: Perfectionism and disordered eating

##### Perfectionism (EDI-P)

Perfectionism was assessed by the 6-item perfectionism scale in the Eating Disorders Inventory (EDI) for children and adolescents [51]. The perfectionism items (EDI-P) are usually rated on a 6-point Likert scale. In the present study, however, the response options were “not true” (scored 0) “sometimes true” (scored 1), and “true” (scored 2) in accordance with the version employed in the youth@hordaland survey [52]. This yields a potential score of 0–12 when the items are summed. Previous research has found that the EDI [53] and EDI-P [54] have satisfactory psychometric properties in similar populations. Cronbach’s $\alpha$ was 0.72 in the present study, indicating acceptable internal consistency.

##### Eating Disturbance Scale (EDS-5)

Symptoms of disordered eating was assessed using the Eating Disturbance Scale (EDS-5) [55]. EDS-5 consists of five questions specifically related to eating, such as comfort eating (item 2) and strict dieting in order to control ones eating habits (item 4). The response options are “not true” (scored 0) “sometimes true” (scored 1), and “true” (scored 2), and the summed scored ranges between 0 and 10. The questionnaire have shown adequate psychometric properties and convergent validity in previous research [55, 56]. Cronbach’s $\alpha$ was 0.78 in the present study, indicating an acceptable internal consistency.

#### Operationalization of EDI-P and EDS-5

For the purposes of the present study, both EDI-P and EDS-5 were used as continuous measures, as well as dichotomous variables, differentiating between low and high scores based on the 90th percentile. The chosen cut-off point is informed by previous research which suggest this to be an adequate delineation for mental health problems [52, 57].

#### Diagnosis of eating disorder

For the participants participating in the study in 2020, self-reported psychiatric diagnoses were available (*n* = 1978) using a pre-defined list adapted to fit this age-group. Initially, the participants had to answer “yes” or “no” to the question “Have you ever received a diagnosis for a mental health problem?”, followed up by a list of 11 possible different diagnoses for those who endorsed the initial question. The list was based on a similar operationalization used in a large population-based studies [58, 59]. The list contained no definition of the included disorders or conditions. For this study, the participants who chose “Eating disorder” (*n* = 36; 1.8%) from the list were identified as having been diagnosed with the condition, and all others were designated as not having received the diagnosis.

### Statistical procedure

First, summary statistics of the included variables for the whole sample were estimated across the median-split of SPAUSCIS and presented in Table 1. For categorical variables, the number and proportions were estimated, and the mean and standard deviation (SD) was estimated for continuous variables. Comparisons across the median-split of SPAUSCIS was done using Pearson’s chi-squared tests for categorical variables, and Wilcoxon rank sum tests were used for continuous variables. Then, two simple linear regression models were estimated using SPAUSCIS as an independent variable and (a) score on perfectionism (EDI-P) and (b) score on disordered eating (EDS-5) as dependent variables, respectively. The scores of the dependent variables were standardized (Z-scored) to ease interpretation of the resulting coefficients. Potential gender-moderation was investigated by entering genderxSPAUSCIS in both models as an interaction term into the model. The interaction term was considered statistically significant with a p-value of < 0.05, and if significant, results from the linear regression model were then presented separately for girls and boys. Linearity of the association between SPAUSCIS and the dependent variables were investigated using restricted cubic splines with four knots. Next, two gender-specific multiple logistic regression models were estimated using the median-split of SPAUSCIS as the main independent variable, and the 90th percentile score on (a) perfectionism (EDI-P) and (b) disordered eating (EDS-5) as dependent variables, respectively. Both models were adjusted for usual amount of social media use and socioeconomic status, and the results are presented as odds ratios with corresponding 95% confidence intervals. The median-split of SPAUSCIS were used in these models for simplicity and ease of interpretability. In post-hoc analyses, we did however, investigate the association between SPAUSCIS as a continuous measure and the 90th percentile score (a) perfectionism (EDI-P) and (b) disordered eating (EDS-5) as dependent variables, respectively. This was done using logistic regression analyses with restricted cubic splines to test for non-linearity. Both these models were adjusted for usual amount of social media use and socioeconomic status, and the results are presented in-text as odds ratios for trends with corresponding 95% confidence intervals. Finally, we investigated the association between the median-split of SPAUSCIS and self-reported eating disorder using simple logistic regression. No adjustments or investigation of potential gender-moderation was included for the latter analyses as the number reporting eating disorder (*n* = 36) limited the statistical precision. Missing data ranged from *n* = 2 (0.1%) to *n* = 55 (1.6%) across analyses, and pairwise deletion was applied to ensure the highest number of observations in each analysis.

## Results

Descriptive statistics of the included variables are presented across the median split of score on SPAUSCIS in Table 1. For all of the included variables, there were significant differences between the SPAUSCIS-groups (all p-values < 0.001). The group with median or above scores on SPAUSCIS were more likely to be girls, more likely to use social media more often and for a longer duration but reported a slightly lower subjective socioeconomic status. Furthermore, they were more likely to report higher scores on perfectionism (EDI-P) and disordered eating (EDS-5).

**Table 1:** Characteristics of included variables across focus on self-presentation

	Self-Presentation and Upward Social Comparison Inclination Scale (SPAUSCIS)		
Characteristic	Below median, *n* = 1,752^1^	Median or above, *n* = 1,672^1^	p-value^2^
Gender			< 0.001
Boys	1,095 (62%)	413 (25%)	
Girls	657 (38%)	1,259 (75%)	
Frequency of social media use			< 0.001
Every day or less	574 (33%)	250 (15%)	
Many times a day	844 (48%)	856 (51%)	
Almost constantly	332 (19%)	566 (34%)	
Duration of social media use			< 0.001
Less than 2 h	647 (37%)	366 (22%)	
2–4 h	637 (37%)	658 (39%)	
> 4–5 h	254 (15%)	360 (22%)	
More than 5 h	205 (12%)	282 (17%)	
Subjective socioeconomic status (0–10)	7.29 (1.77)	7.01 (1.78)	< 0.001
Eating Disorders Inventory, perfectionism (EDI-P)	4.92 (2.61)	5.87 (2.65)	< 0.001
EDI-P, 90th percentile			< 0.001
Below 90th	1,661 (95%)	1,507 (90%)	
90th or above	87 (5.0%)	163 (9.8%)	
Eating Disturbance Scale (EDS-5)	2.17 (2.22)	3.81 (2.77)	< 0.001
EDS-5, 90th percentile			< 0.001
Below 90th	1,637 (94%)	1,328 (80%)	
90th or above	110 (6.3%)	342 (20%)	

Note: SPAUSCIS (Self-Presentation and Upward Social Comparison Inclination Scale). Missing data ranged from *n* = 2 (0.1%) to *n* = 15 (0.4%) for individual variables

^1^n (%); Mean (SD)

^2^Pearson’s Chi-squared test; Wilcoxon rank sum test

Results from gender-specific multiple logistic regression models with median-split of SPAUSCIS as dependent variable, and the 90th percentile score on (a) perfectionism (EDI-P) and (b) disordered eating (EDS-5) as dependent variables is presented in Table 2. For boys and girls, scoring on or above the median on SPAUSCIS was associated with increased odds for both dependent variables. For both perfectionism and disordered eating, the models are adjusted for social media use and socioeconomic status. In the post-hoc analyses using SPAUSCIS as continuous variable, the odds ratios (OR) in relation to perfectionism were 1.88 (95% CI 1.43–2.47, *p* < 0.001) and 1.77 (95% CI 1.44–2.17, *p* < 0.001) for boys and girls, respectively. For disordered eating, the corresponding ORs were 1.94 (95% CI 1.40–2.68, *p* < 0.001) for boys and 2.00 (95% CI 1.72–2.32, *p* < 0.001) for girls. Using restricted cubic splines, we did not find evidence for non-linearity in the post-hoc analyses.

**Table 2:** Association between focus on self-presentation and perfectionism and disordered eating. Logistic regression, stratified by gender

		Boys				Girls			
Group	Characteristic	n	OR^1^	95% CI^1^	p-value	n	OR^1^	95% CI^1^	p-value
EDI-P, 90th percentile	SPAUSCIS, median split	1,478				1,892			
	Below median		—	—			—	—	
	Median or above		2.27	1.46, 3.50	< 0.001		1.71	1.16, 2.57	0.008
EDS-5, 90th percentile	SPAUSCIS, median split	1,477				1,892			
	Below median		—	—			—	—	
	Median or above		2.58	1.51, 4.42	< 0.001		2.14	1.64, 2.84	< 0.001

Note: Adjusted for usual amount of social media use and socioeconomic status; SPAUSCIS: Self-Presentation and Upward Social Comparison Inclination Scale; EDI-P: Eating Disorders Inventory-Perfectionism; EDS-5: Eating Disturbance Scale-5

^1^OR = Odds Ratio, CI = Confidence Interval

There was a significantly higher odds of reporting being diagnosed with an eating disorder among those scoring median or above on SPAUSCIS (crude OR 3.32; 95% CI 1.58–7.84; *p* = 0.003).

**Fig. 1:**
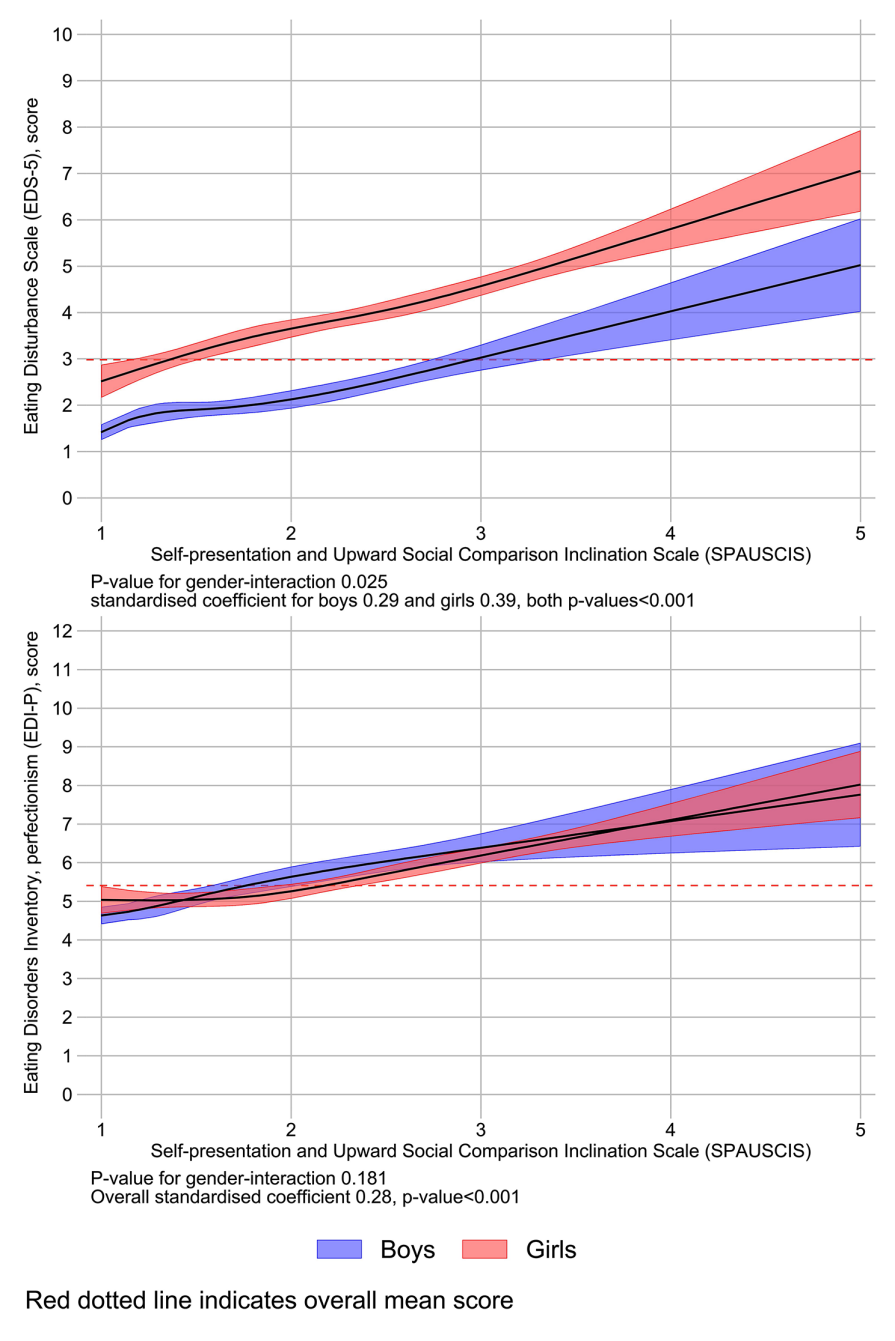
Association between focus on self-presentation and perfectionism and disordered eating. Linear regressions with restricted cubic splines. Note: Figure 1: SPAUSCIS: Self-Presentation and Upward Social Comparison Inclination Scale; EDI-P: Eating Disorders Inventory-Perfectionism; EDS-5: Eating Disturbance Scale-5

Figure 1 presents findings from linear regression models with mean score on SPAUSCIS as the independent variable and the standardized (Z-scored) score on (a) perfectionism (EDI-P) and (b) disordered eating (EDS-5) as dependent variables. For both dependent variables, a potential gender moderation of the association with SPAUSCIS was investigated, and potential non-linearity was investigated using restricted cubic splines with four knots. For disordered eating, a significant gender moderation was found, and the association was stronger for girls than boys. For perfectionism, no evidence for a gender moderation was found. For both dependent variables there was a significant linear association with self-presentation equal to a low-to-moderate effect size.

## Discussion

### Overall findings

In the present study we investigated the potential association between focus on self-presentation and upward social comparison on social media, and perfectionism and disordered eating. As hypothesized, we found evidence for consistent positive associations. Increased focus on self-presentation and upward social comparison was associated with increased levels of both perfectionism and disordered eating with a small-to-medium effect size. For perfectionism, the associations were similar for both boys and girls, while we found evidence of a gender moderation for disordered eating. Specifically, the association with disordered eating was somewhat stronger for girls compared to boys. For self-reported eating disorder, we also found a positive association with focus on self-presentation and upward social comparison. Focusing on how the adolescents relate to self-presentation on social media, the study gives new insight into important aspects of usage patterns of social media. It also provides new insight into potential gender differences in focus on self-presentation and upward social comparison on social media, and social media´s potential role in development of disordered eating. These findings are pertinent in a public health perspective and may help to inform efforts to mitigate these potential negative effects.

### Relation to previous perspectives and findings

Our findings are consistent with the Tripartite Influence Model, as our study revealed positive associations between focus on self-presentation and upward social comparison on social media, and both perfectionism and disordered eating. Individuals who focus on self-presentation and upward social comparison may be more susceptible to sociocultural pressures which may lead to a strive for perfection and conforming to unhealthy body ideals. Our findings underscore the potential role of sociocultural pressures in shaping body image dissatisfaction and disordered eating behaviors. Specifically, the positive association between focus on self-presentation on social media and perfectionism may have several explanations. Curran & Hill [33] argue that the increase in perfectionistic traits among young adults may be due to a response to cultural changes towards a more individualistic and competitive culture in Western societies. As social media is an important part of adolescents’ and young peoples’ lives, it is likely that the perfectionistic tendencies will affect self-presentation on these platforms as well. Curran & Hill [33] also suggest that the increase in perfectionism among young adults may be due to their perception of increased demands from the social environment. Self-presenting in a socially desirable way in general, and on social media specifically, may be a way to ensure social acceptance from peers. They further hypothesize that the fear of losing acceptance may increase perfectionistic traits [33]. Hence, increased perfectionism may be the reason for stronger focus on self-presentation on social media. However, since we cannot interpret the direction of the association from this study, focus on self-presentation may also increase adolescents’ perfectionistic tendency. As perfectionism is a personality trait that largely establishes during adolescence, it may be that the increased opportunity to self-present on social media, and thus focus on self-presentation, makes adolescents more susceptible for developing perfectionistic traits.

There is a lack of research on the relationship between focus on self-presentation on social media and disordered eating. Most of the research investigating this relationship have looked at being exposed to appearance-related self-presentation on social media and body dissatisfaction and disordered eating [24, 25, 43], in addition to self-presentation behavior [13, 14, 45], not the relationship between a person’s *focus* on self-presentation on social media and disordered eating. Our results indicate a positive relationship between focus on self-presentation on social media and disordered eating. Highly visual social media platforms that expose adolescents to “perfect” bodies through others’ self-presentation may constitute an important source of such exposure. Previous findings support that being exposed to body ideals, may lead to internalization of these ideals among adolescents [25, 45, 46]. Other findings also report that upward social comparison may be a potential consequence of the exposure to others’ “perfect” appearance related self-presentation [24, 31, 60], leading to body dissatisfaction [30]. Subsequently, some adolescents may be more preoccupied with eating, weight, body shape, and muscularity. This preoccupation could serve as a mitigation strategy to reduce the discrepancy between the adolescent’s perceived appearance and the ideal body and appearance of the reference person. Thereby reducing the negative body image and negative feelings produced from the upward social comparison.

Another explanation may be that adolescents with disordered eating already are more preoccupied than other adolescents with how they appear to others. Social media is an apt arena to self-present in an appearance-related and desired way, and could elicit wanted feedback from others through likes and comments. This may further reinforce the focus on self-presentation. A third potential explanation for this relationship is perfectionism as a conceivable mediating factor. As perfectionistic self-presentation can be understood as a maladaptive self-presentation style [34], perfectionism may lead to a strict view of what constitute a good-enough self-presentation. This may as well include the adolescent’s expectations and demands to their own body as thin or muscular, hence increasing the standards of flawlessness in their own appearance-related self-presentation on social media. If these expectations are too rigid, it might for some adolescents be a contributing cause in the development of disordered eating.

In relation to the association between focus on self-presentation on social media and disordered eating we found a stronger association for girls than boys. Hjetland et al. [61] found significant gender differences in how adolescents related to self-presentation on social media. Girls reported that they invested more time and energy on the content of their own social media posts. They used more filters to look better at least sometimes and reported feeling less satisfied with themselves because of other peoples’ social media posts. Girls also tended to ascribe more importance to the feedback they got on social media than boys. In general, the report showed that social media played a bigger part in the girls’ lives than the boys’, and that the girls placed more importance on what is happening on social media [61]. Hence, more importance placed on self-presentation on social media among girls, and social media playing a more important role in girls’ lives, may increase the focus on self-presenting in an ideal way, in addition to being stronger underlying causes in development of eating disorders for girls than for boys.

There may as well be other explanations for the gender difference we found. The objectification theory [62], suggests that women’s bodies are more often looked at, evaluated and potentially sexually objectified. Fredrickson & Roberts [62] further argue that these views make women internalize the observer’s perspective of themselves, and to some degree also socialize women to treat themselves as objects for the pleasure of others. The emphasis put on girls’ and women’s physical appearance, in particular, is well established in our culture [60]. Through social media’s feedback mechanisms, girls may be more encouraged than boys to self-present in an objectifying way.

Social comparison theory [26], and especially upward social comparison, is another possible explanation for the gender difference between focus on self-presentation on social media and disordered eating. Strahan et al. [60] found that when describing their physical appearance, women used significantly more upward social comparisons than downward social comparisons. Men, on the other hand, made more downward comparisons than upward. This tendency was not seen when women and men described other personal characteristics like social skills. For women, they also found that the more upward social comparison they made, the more negative statements they made about their body [60]. They proposed that ubiquitous appearance norms, mostly applying to women, disrupted strong self-enhancement behaviors [60]. Fardouly et al. [24] also found that women relied on upward social comparisons when comparing their appearances, and that doing this on social media was associated with more body dissatisfaction than in person. A proposed explanation for this is that women may experience a stronger discrepancy between themselves and women they see on social media compared to women they see in person [24].

Previous research on self-presentation behaviors has primarily focused on appearance-related self-presentation and upward social comparison [e.g. 24] and associated risk among girls, such as body dissatisfaction [13, 14, 30], thin ideal internalization and disordered eating behavior [25, 44]. However, it is important to recognize that boys may also be affected by these issues, and a study showed that body dissatisfaction affected boys’ risk of engaging in disordered eating behaviors [63]. The current body ideals for boys emphasize muscularity [64], and Eisenberg et al. [65] found that muscle-enhancing behaviors are common among American adolescents, including both boys and girls. This were behaviors like dieting, exercising, and taking protein supplements or steroids, with the aim of increasing muscle size or tone. However, most of the behaviors measured were significantly more common among boys [65], and Compte et al.’s [64] investigation of muscle dysmorphia among young adult men indicated a prevalence of almost 7%. Hence, another explanation for the gender difference we found, may be that the EDS-5-questionnaire does not identify symptoms of drive for muscularity or muscle dysmorphia. In fact, muscle dysmorphia seems to be more of a concern than thinness and weight loss among boys [64]. The EDS-5 measures of symptoms of disordered eating are linked to preoccupation about weight loss, body shape and drive for thinness [55], and may therefore not fully capture the range of body image concerns among boys.

### Implications

The present results demonstrate the need to address focus on self-presentation and upward social comparison on social media as potentially important factors for adolescents’ mental health. As such, promoting a healthy use of social media could be established through a focus on increasing adolescents’ ability to reflect on and think critically about self-presentation and upward social comparison on social media. Our results indicate a need for targeted interventions to promote healthy social media use and enhance adolescents’ critical thinking about self-presentation and underscores the urgency of public health initiatives. One public health approach would be to equip adolescents with critical thinking skills to navigate social media mindfully. In relation to appearance-related ideals, educational programs should address the unrealistic standards perpetuated online, while fostering resilience and promoting positive self-image. Educational programs and social media literacy programs in school have been suggested to increase adolescents’ reflections about their own and others social media use [42, 66, 67]. Gordon et al. [42, 67] introduced a four-lesson social media literacy program in a junior high school that aimed to decrease body dissatisfaction, dietary restraints and focus on increased muscles among young adolescents. They found only a small effect of the intervention. The intervention did not focus on self-presentation and based on results from this study and previous research [e.g. 12, 27], this would be an important topic to address for future interventions. Also, previous results suggest that interventions led by individuals who already have an established relationship with the adolescents and are familiar with their needs help facilitate discussions among the adolescents [42], and improve intervention outcomes. Teachers could therefore be considered effective social media educators, especially if social media literacy could be integrated in existing school subjects.

A study of university students showed that women who had a higher internalization of the thin-ideal, were more vulnerable to disordered body image and hence to appearance exposure in media [68]. They also found that body appreciation protected women from negative effects of the exposure [68]. Thus, developing social media literacy programs specifically focusing on the effects of self-presentation and upward social comparison could be an important target for interventions, and possibly reduce focus on self-presentation. Research [69] also suggest that increasing self-compassion is a useful strategy to prevent perfectionistic self-presentation on social media. As perfectionistic self-presentation is related to lower subjective well-being [35], this may also be a topic to address in an intervention aiming to reduce focus on self-presentation and upward social comparison on social media.

While our study adds to the knowledge base, future research should investigate the concept of self-presentation on social media more closely. It will be important to examine if different ways of self-presentation vary from each other. Previous research has investigated how people self-present, especially through the use of selfies [e.g. 70, 71], and further research should investigate if taking pictures of oneself and posting them is dissimilar from other ways of self-presentation on social media when considering its association to mental health among adolescents. SPAUSCIS consist of only one item asking about specific ways of self-presenting (“I retouch images of myself to look better before I post them on social media”), thus future research on other ways of self-presenting behaviors should include self-presentation for example through pictures of other aspect of the adolescents’ life, like friends or hobbies, or through text only. Investigating focus on self-presentation on social media, perfectionism and disordered eating among younger adolescents than we included in our study will be important as the use of social media starts early and as disordered eating often emerges in adolescence [72]. Understanding at what age focus on self-presentation becomes more prominent for adolescents’ and potential gender differences regarding this, may also be important to pinpoint intervention opportunities.

### Strengths and limitations

A major strength of the present study is that it is the first study to investigate the relationship between focus on self-presentation on social media, perfectionism and disordered eating. So far, the research on this has focused on self-presentation behaviors [e.g. 13, 14, 30, 45] in addition to being exposed to others*’* (perfect) self-presentations and the prevailing body ideals [e.g. 24, 25, 43]. To our knowledge no previous study has examined the association between focus on self-presentation and perfectionism and disordered eating. In addition, the scales used in this study are well-established [54–56]. Also, the items of SPAUSCIS were derived from focus-group interviews with adolescents [23], which make them relevant for adolescents’ experiences related to self-presentation and social comparison on social media. Some limitations are also worth mentioning. The study is cross-sectional, thus we cannot determine causality between the investigated factors and mental health. Despite the sample being large, it is limited to high schools in Bergen, Norway. Consequently, the results may not be generalizable to other countries or cultures. Also, the participation rate was moderate (53% and 35%), which may impact the validity of our findings. However, associations are less vulnerable to bias caused by low participation rates than prevalence estimates [73]. Another limitation is that SPAUSCIS in this study does not differentiate between various methods of self-presentation. Consequently, we cannot conclude from this study whether specific types of self-presentation, such as taking selfies versus posting pictures of hobbies, have the same impact on perfectionism, eating disorders or disordered eating. Also, the use of self-reported amount of social media use has been shown to be biased in previous research and is not likely to be an accurate measure of actual use [74]. This may have impacted our ability to effectively account for the confounding effect of social media use. And finally, although EDS-5 is a well-established and validated measurement, the questionnaire does not cover specific symptoms of drive for muscularity and muscle dysmorphia.

## Conclusions

While previous studies have focused on self-presentation behaviors, this study found that focus on self-presentation and upward social comparison on social media is positively associated with both perfectionism and disordered eating, as well as self-reported eating disorders among adolescents. As such, promoting a healthy use of social media could be established through increasing adolescents’ ability to reflect on and think critically about self-presentation and upward social comparison on social media. Our results underscore the importance of targeted public health interventions to promote awareness and healthy social media use among adolescents, emphasizing the need for educational programs that address focus on self-presentation, unrealistic appearance-related ideals and foster resilience and positive self-image.

## Data Availability

Explicit consent from the participant is required by the Norwegian Health research legislation and the Norwegian Ethics committees in order to transfer health research data outside of Norway. Ethics approval for this was also dependent on storing the research data on secure storage facilities located at the Norwegian Institute of Public Health, which prevent the authors from providing the data as supplementary information. Request to access these datasets should be directed to jens.christoffer.skogen@fhi.no.

## Declarations

Ethics approval and consent to participate:
